# Maternal Inheritance of a Recessive RBP4 Defect in Canine Congenital Eye Disease

**DOI:** 10.1016/j.celrep.2018.04.118

**Published:** 2018-05-29

**Authors:** Maria Kaukonen, Sean Woods, Saija Ahonen, Seppo Lemberg, Maarit Hellman, Marjo K. Hytönen, Perttu Permi, Tom Glaser, Hannes Lohi

**Affiliations:** 1 Department of Veterinary Biosciences, University of Helsinki, 00014 Helsinki, Finland; 2 Research Programs Unit, Molecular Neurology, University of Helsinki, 00014 Helsinki, Finland; 3 The Folkhälsan Institute of Genetics, 00290 Helsinki, Finland; 4 Department of Cell Biology and Human Anatomy, University of California, Davis School of Medicine, Davis, CA 95616, USA; 5 Department of Eye Diseases, Helsinki University Hospital, 00029 The Hospital District of Helsinki and Uusimaa, Finland; 6 Department of Chemistry, Nanoscience Center, University of Jyväskylä, 40014 Jyväskylä, Finland; 7 Department of Biological and Environmental Science, Nanoscience Center, University of Jyväskylä, 40014 Jyväskylä, Finland

## Abstract

Maternally skewed transmission of traits has been associated with genomic imprinting and oocyte-derived mRNA. We report canine congenital eye malformations, caused by an amino acid deletion (K12del) near the N terminus of retinol-binding protein (RBP4). The disease is only expressed when both dam and offspring are deletion homozygotes. RBP carries vitamin A (retinol) from hepatic stores to peripheral tissues, including the placenta and developing eye, where it is required to synthesize retinoic acid. Gestational vitamin A deficiency is a known risk factor for ocular birth defects. The K12del mutation disrupts RBP folding in vivo, decreasing its secretion from hepatocytes to serum. The maternal penetrance effect arises from an impairment in the sequential transfer of retinol across the placenta, via RBP encoded by maternal and fetal genomes. Our results demonstrate a mode of recessive maternal inheritance, with a physiological basis, and they extend previous observations on dominant-negative RBP4 alleles in humans.

## In Brief

Maternal inheritance distinctive from imprinting and oocyte-derived mRNA mechanisms has been regarded as a rare exception unique to humans. Kaukonen et al. describe a canine model with a recessive maternally transmitted RBP4 defect, suggesting that this mechanism is more common in developmental defects.

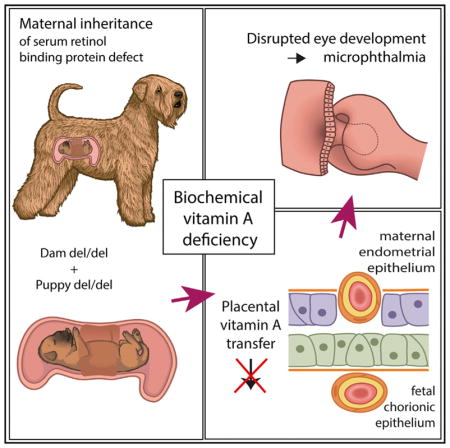

## INTRODUCTION

The microphthalmia, anophthalmia, and coloboma (MAC) spectrum of congenital eye malformations are important causes of childhood blindness (Hornby et al., 2000). Anophthalmia refers to the complete absence and microphthalmia to reduced size of the ocular globe. Colobomas are notch-like defects in the iris, chorioretina, and/or optic nerve head that result from incomplete closure of the axial optic fissure during development (Onwochei et al., 2000). MAC disease has a worldwide incidence of 1 per 5,300 live births (Morrison et al., 2002). Most cases are isolated, with defects limited to the eye, but in one-third of patients the eye malformations occur as part of a syndrome (Verma and Fitzpatrick, 2007). Potential mechanisms include primary failure of optic vesicle growth, optic cup invagination or lens induction, or secondary degeneration of optic anlagen in utero (Graw, 2003). In most cases, the etiology is unknown. Recent reports implicate *SOX2*, *OTX2*, *STRA6*, and *PAX6* (Fantes et al., 2003; Ragge et al., 2005; Pasutto et al., 2007; Glaser et al., 1994), with dominant *SOX2* loss-of-function alleles being the most common single-gene defect (Gerth-Kahlert et al., 2013). Apart from gene mutations, various environmental risk factors have been reported for human MAC disease, most notably vitamin A deficiency (VAD) (Hornby et al., 2002). Vitamin A (retinol) is a substrate for synthesis of retinoic acid (RA), a potent paracrine-signaling molecule needed for proper development of the vertebrate eye and other tissues (Hale, 1935; See and Clagett-Dame, 2009). The eye is most sensitive among organs to reduced RA levels during embryogenesis.

In recent years, the domestic dog has emerged as a powerful model for study of simple and complex mammalian traits, due to its unique genetic architecture and abundant genomic tools (Lindblad-Toh et al., 2005). The canine eye more closely resembles the human eye, anatomically and physiologically, than do mouse or rabbit eyes and spontaneous hereditary eye diseases are common (Vaquer et al., 2013). Microphthalmia has been reported in several dog breeds, including Irish soft-coated wheaten terriers (ISCWTs), in which microphthalmia, retinal coloboma, hypoplasia of the choroid, and severe visceral malformations were reported (Van der Woerdt et al., 1995). The ocular phenotypes resemble the most severe features of collie eye anomaly (CEA), but they are genetically distinct (Parker et al., 2007).

In this study, we report a retinol-binding protein (*RBP4*) defect in a canine developmental eye disease; characterize its clinical, genetic, and biochemical properties; and consider the physiological implications of this unique recessive maternal penetrance effect.

## RESULTS

### Microphthalmia and Other Developmental Eye Defects in ISCWTs

**Figure 1:**
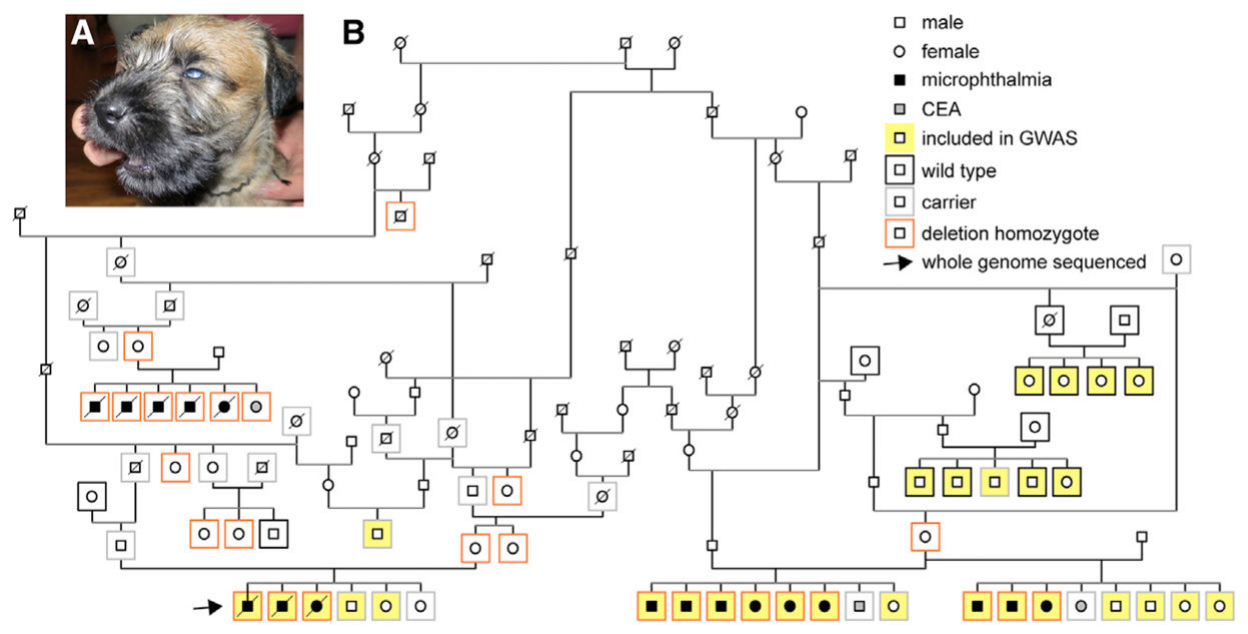
Autosomal Recessive Micro-phthalmia in ISCWT Dogs (A) External eye phenotype, showing microphthalmia. (B) Pedigree diagram showing lineage relationships among diseased dogs, clinical eye findings, *RBP4* genotypes, and the maternal penetrance effect. Phenotypes and confirmed DNA genotypes (n = 67) are indicated by inner and outer symbols, respectively, as described in the inset legend. Dogs included in the GWAS are highlighted in yellow (12 cases and 17 controls). All 17 microphthalmic dogs (■ ●) and their dams are *RBP4* deletion homozygotes (del/del). One deletion homozygote with a homozygous dam had eyes of normal size, with chorioretinal hypoplasia. Ten dogs without microphthalmia (□ ○) were also genotyped as homozygous (del/del), but they had heterozygous (del/+, n = 9) or untyped (del/−, n = 1) dams. See also Figure S2 and Table S1.

An ISCWT breeder in Finland contacted us in 2011 after noticing abnormally small eyes in three pups in a litter of six. Eye exams before 10 weeks of age revealed bilateral microphthalmia with scleral folding, chorioretinal hypoplasia, and retinal colobomas in the affected dogs (Figure 1A; Table S1). A second affected litter of eight was subsequently born in Poland. Eye exams confirmed bilateral microphthalmia in six pups and chorioretinal hypoplasia in one pup and unilateral retinal coloboma. Then 2 years later, another litter was born to the same dam with a different sire. Three of eight pups had bilateral microphthalmia, chorioretinal hypoplasia, and retinal colobomas; one had a unilateral flat optic nerve head; and four were unaffected. In a fourth litter, born in the Czech Republic, five of six pups had bilateral microphthalmia and one had chorioretinal hypoplasia. The dam and sire of each litter had normal eye exams. The dams were fed high-quality commercial chow and no abnormalities were noted during gestation.

For genetic analysis, our inclusion criteria for subject dogs (cases) was bilateral microphthalmia (n = 17, with 11 males and 6 females). Normal control dogs (n = 23), ascertained from the same large ISCWT pedigree, were carefully examined by a veterinary ophthalmologist before 10 weeks of age, as tapetal pigmentation in older dogs can mask milder forms of chorioretinal hypoplasia (Bjerkås, 1991).

### Genetic Analysis Reveals an In-Frame 3-bp Deletion in RBP4

**Figure 2:**
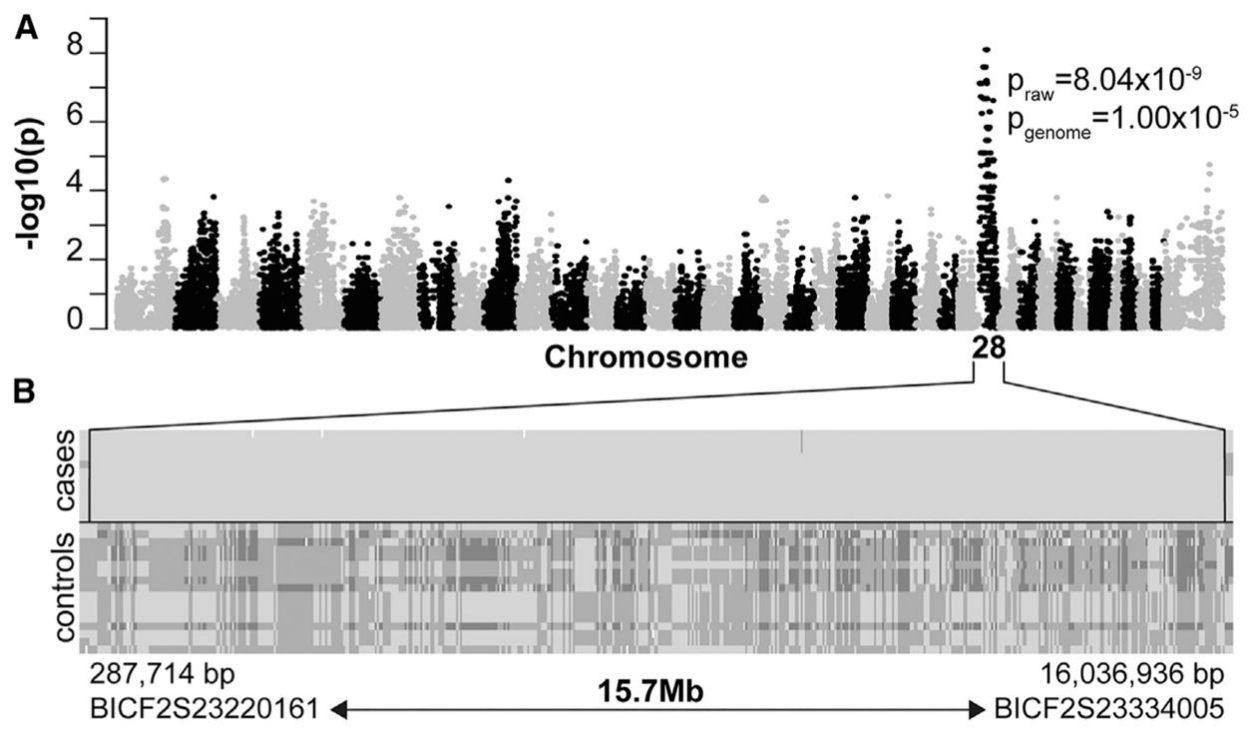
Mapping the ISCWT Microphthalmia Locus (A) Summary of GWAS data. Manhattan plot of a genome-wide case-control association analysis, with 12 cases and 17 controls, shows localization of microphthalmia trait on CFA28, with a corrected probability value *P*_genome_ = 1.00 × 10^−5^. (B) Genotype data showing a shared haplotype block in microphthalmic dogs (cases). Each row represents a single animal, with the genotypes at each SNP locus (columns) indicated by dark (AA), intermediate (AB), or light (BB) shading, in relation to the affected haplotype (BB). Every affected dog is homozygous within this segment. The 15.7-Mb critical region spans approximately 38% of CFA28, near the centromere, and it is delimited by SNP markers BICF2S23220161 (centromeric) and BICF2S23334005 (telomeric). See also Figure S1.

The four affected litters are related in a single pedigree, with a transmission pattern suggesting an autosomal recessive mode of inheritance as several affected pups in different litters were born to unaffected parents (Figure 1B). To map the disease locus, we performed a genome-wide association study (GWAS) with 12 cases, 17 controls, and 172,963 SNP markers. Statistical analysis of genotype data by PLINK indicated a 15.7-Mb critical region on canine chromosome 28 (*p*_raw_ = 8.04 × 10^−9^, *p*_genome_ = 1.00 × 10^−5^), spanning nucleotides 287,714 to 16,036,936 bp (CanFam 3.1), in which all cases shared a single homozygous haplotype block (Figure 2). The localization was confirmed by GenABEL analysis, using a full genomic kinship matrix to adjust population structure and mixed model approximation (Figure S1).

**Figure 3:**
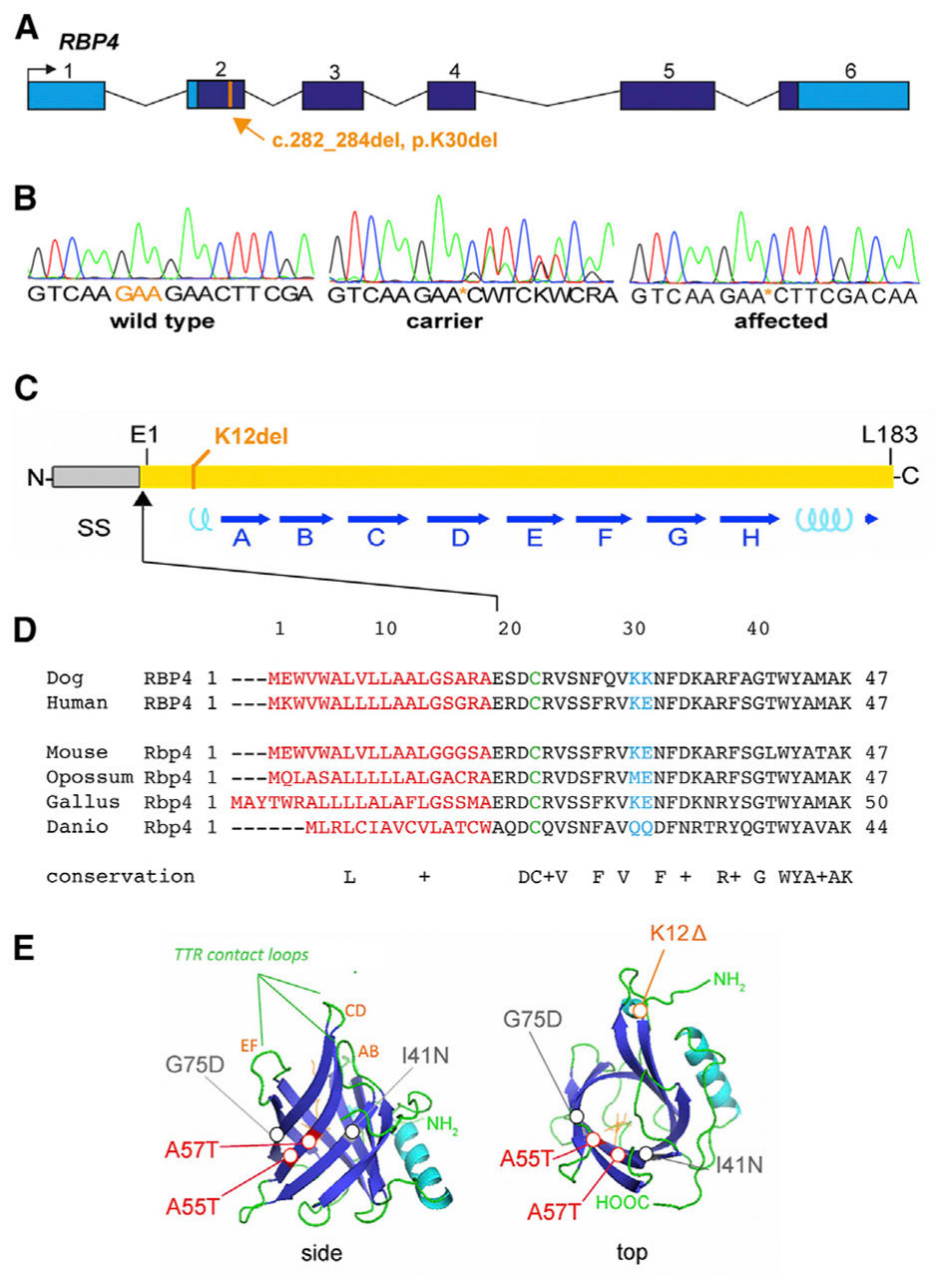
Pathogenic *RBP4* Deletion (A) p.K30del mutation. Genomic map shows the solitary coding variant identified in the critical region by whole genome sequencing, a 3-bp deletion in exon 2 that removes lysine codon 30 from the RBP precursor. This residue corresponds to K12 in the mature polypeptide, after signal peptide cleavage. Coding (dark blue) and UTR sequences (light blue) are indicated. (B) Sanger chromatograms showing the DNA sequence of PCR products spanning the *RBP4* deletion in wild-type (WT), carrier, and affected dogs. The deletion removes one of two tandem lysine codons (AAG). (C) Linear diagram of RBP showing the signal peptide (SS); 8 antiparallel β sheets (A–H, blue arrows), which form the ligand barrel; 2 short α-helical segments (cyan coils); 3 cysteine disulfide bonds, which stabilize the tertiary structure; and the mutated K12 residue. (D) Alignment of vertebrate RBP sequences showing evolutionary conservation of K12 among eutherians. The signal peptide (red), tandem lysines (K12–K13, blue), and disulfide-linked cysteine (C4, green) are indicated. The N-terminal segment preceding the β-barrel (10 of 21 residues) is highly charged. (E) Tertiary structure of canine RBP (ribbon views), modeled from human *apo* (1RBP) and *holo* (1BRQ) RBP X-ray data (Cowan et al., 1990; Zanotti et al., 1993a, 1993b), showing K12 near the N terminus, within an α-helical region. By shortening this segment, K12del may limit apposition of C4 and C160 side groups in the ER, preventing formation of one disulfide bond *in vivo* and, consequently, destabilizing the protein. See also Table S2.

To identify the causative variant, we sequenced the entire genome of one affected dog. A total of 470,800,949 reads were collected, of which 98.7% were mapped to the reference genome (CanFam 3.1). The mean read depth was 28.7× and 98.3% of mapped reads had >10× coverage. We identified 6,497,411 homozygous variants compared to the reference sequence, and 37,291 of these remained after filtering variants from 342 control dogs of breeds that lack the studied phenotype (Table S2). Among the remaining variants, 81 were exonic, but only one of these was located in the CFA28 critical region. This variant is a 3-bp deletion (c.282_284del) in the gene encoding *RBP4* gene, resulting in the loss of a single lysine (AAG codon) near the RBP amino terminus (p.K30del), in a charged segment preceding the lipocalin β-barrel domain (Figure 3). This is the 12^th^ amino acid in the mature protein (K12del), after cleavage of the signal peptide, and it is highly conserved among vertebrates. The secreted portions of dog and human RBP are the same length (183 amino acids) and have 94.5% sequence identity.

### Maternal Inheritance Effect

To confirm that the *RBP4* variant segregates with the disease trait, we genotyped all available dogs (n = 46) from affected litters and their close relatives (Figure 1B). As expected, the 17 cases were homozygous for the K12 deletion, and the 23 clinically confirmed controls were wild-type (WT) (+/+) or heterozygous (del/+). However, the three dams of the four affected litters were also homozygous for the deletion yet had normal eye exams. Notably, *their* dams were heterozygous. These results suggest a recessive mode of inheritance with reduced penetrance and a potential maternal genotype effect (Figure 1B).

To further evaluate the maternal effect on inheritance, we genotyped all available ISCWT samples in our biobank (n = 248). This analysis revealed 185 WT dogs (74.6%), 55 carriers (22.2%), and 8 homozygotes (3.2%), consistent with Hardy-Weinberg equilibrium (p = 0.32, χ^2^ test, *df* = 2). Among these eight new K12del homozygotes, three had normal fundus eye exams as adults, four had normal general exams with no clinically apparent microphthalmia, and one suffered from chorioretinal hypoplasia. Maternal genotypes are known for seven of these dogs; in each case, the dam was heterozygous, except for the dog with chorioretinal hypoplasia, whose dam was an *RBP4* deletion homozygote. These data demonstrate a striking maternal transmission effect on inheritance (Table 1; p < 10^−4^, Fisher’s exact test, *df* = 1). Accordingly, the microphthalmia trait is manifest only when *both* dam and offspring are homozygous for the deletion (17/18). If the dam is heterozygous, her homozygous offspring have grossly normal eye anatomy (9/9). The penetrance of the microphthalmia trait in del/del dogs is thus 94% or 0%, respectively, depending on the dam genotype.

Because some human *RBP4* alleles have dominant phenotypes (Chou et al., 2015), we investigated the clinical status of del/+ carriers in detail. Among 71 heterozygotes in our study cohort, 46 had thorough eye exams. Dam genotypes, determined for 37 of these 46 carriers, were as follows: 14 homozygous (del/del), 11 heterozygous (del/+), and 12 WT (+/+). Two carriers did have CEA-like findings (chorioretinal hypoplasia) but no microphthalmia. These 2 dogs were littermates of affected pups (Figure 1B) and their dams were deletion homozygotes. All other del/+ carriers examined were normal. In particular, 8 of these 12 unaffected carriers born to del/del dams had fundus exams before 10 weeks of age to reliably assess choroidal anatomy (Bjerkås, 1991).

To exclude mild retinal pathology in dogs born to heterozygous dams, we performed OCT (optical coherence tomography) imaging on 11 ISCWTs with different genotypes (3 +/+, 4 del/+, and 4 del/del). Whole retinal thickness (WRT) and photoreceptor layer thickness (PRT) were within normal limits in every dog, and no statistically significant difference was found between genotype groups (Figure S2). Thus, for offspring to manifest eye disease, the dam must be an *RBP4* deletion homozygote, with phenotypic severity depending on the offspring genotype (p < 10^−6^, Fisher’s exact test, *df* = 1). Deletion homozygotes had microphthalmia with nearly complete penetrance (17/18) or chorioretinal hypoplasia (1/18), whereas heterozygotes had a milder condition, such as chorioretinal hypoplasia, with low penetrance (2/14).

### Dose-Dependent Decrease in Serum RBP and Vitamin A Levels

**Figure 4:**
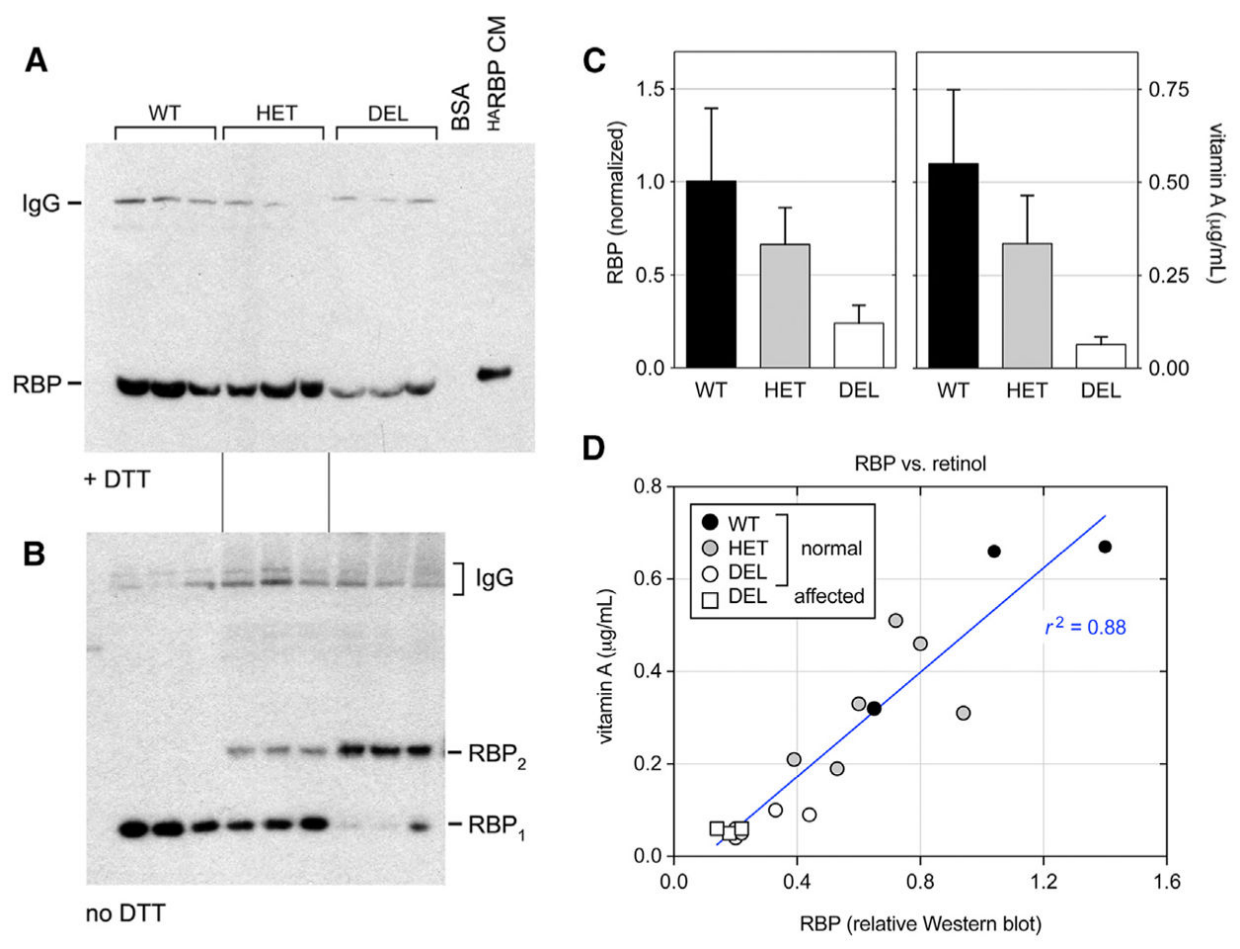
Serum RBP and Vitamin A Analysis (A) ECL western blot (reducing conditions) showing dog RBP monomers (21 kDa) with human ^HA^RBP (22 kDa) as a positive control (HeLa-CM). The higher MW signal is dog IgG (150 kDa), which cross-reacts with the secondary reagent (donkey anti-rabbit IgG) and is relatively resistant to DTT (Singh and Whitesides, 1994). (B) Parallel blot (non-reducing conditions) showing RBP monomers (WT) and homodimers (K12del mutant). Both species are present in heterozygotes, in roughly a 4:1 molar ratio. (C) Histograms comparing immunoreactive serum RBP and vitamin A (mean ± SD) versus genotype. There is a linear dosage relationship, with heterozygotes having an intermediate level of RBP (0.66 ± 0.20) that is close to the arithmetic mean of del/del and +/+ samples (0.62 ± 0.22). (D) Scatterplot comparing serum vitamin A (μg/mL, ordinate) and relative RBP (abscissa) for all samples, including WT (black), HET (gray), and DEL (white) dogs with normal eyes (circles) and microphthalmic DEL dogs (white squares). The values are well correlated (p < 0.0001, *r*^2^ = 0.88); however, the regression line is shifted rightward from the origin, indicating that the K12del protein binds less retinol *in vivo* than WT. The homozygous DEL samples have similar RBP and vitamin A levels, regardless of phenotype (one-way ANOVA, p = 0.18 for RBP and p = 0.45 for vitamin A, comparing 5 normal and 3 microphthalmic dogs). See also Tables S1 and S3.

RBP circulates in blood and transports vitamin A from hepatic stores to peripheral tissues, such as the developing eye. In principle, the K12 deletion, near the ligand-binding domain (Figures 3C–3E) may disrupt RBP folding, stability, or secretion; retinol-binding activity; and/or interaction with the STRA6 receptor. To investigate stability and retinol-binding effects, we measured serum RBP and vitamin A levels in 17 adult ISCWTs, including 8 deletion homozygotes (3 with microphthalmia), 6 del/+ carriers, and 3 WT dogs (Figure 4). Serum albumin and total protein were assayed in parallel as a control. RBP levels were assessed by western analysis, following denaturing gel electrophoresis (SDS-PAGE) under reducing conditions, and were normalized to wild-type. Relative RBP levels (±SD) were roughly halved in heterozygotes (0.66 ± 0.20) and greatly reduced in homozygotes (0.24 ± 0.10) compared to WT dogs (1.00 ± 0.39). The mutant protein is thus poorly secreted or rapidly cleared from the bloodstream.

To assess the structure of circulating canine K12del RBP, we performed western analysis on serum samples under non-reducing conditions (Figure 4B). To maximize exposure of epitopes in native globular RBP after electrophoresis, SDS-PAGE gels were treated with β-mercaptoethanol (βME) before transfer (Zetterström et al., 2007). In these experiments, the K12del protein migrated as an apparent homodimer (42 kDa) in homozygote sera, with little or no monomeric RBP. Presumably, the K12del RBP variant folds abnormally in the hepatic endoplasmic reticulum (ER) of mutant dogs, leading to the formation of intermolecular disulfide bonds, which allows progression of the mutant RBP to the Golgi compartment (Kaji and Lodish, 1993). The mutant dimers were more antigenic than wild-type (WT) monomers in non-reducing western blots, reflecting their partially unfolded status *in vivo* (Figure S3). Consequently, the dimer fraction of serum RBP was determined following in-gel reduction. In heterozygous dogs, the ratio of dimers to monomers was 0.23 ± 0.04, consistent with the overall decrease in serum RBP (Figure 4A). These data, and the linear relationship between genotype and total RBP levels (Figure 4C), indicate that the K12del protein does not significantly dimerize with WT RBP or interfere with its secretion *in vivo*.

Vitamin A levels (±SD) were severely reduced in all deletion homozygotes (0.06 ± 0.02 mg/L), compared to WT (0.55 ± 0.20 mg/L) and the normal canine reference range (0.3–1.3 mg/L), regardless of phenotype (p < 0.0001; Figures 4C and 4D). Vitamin A levels in carriers were 0.34 ± 0.13 mg/L, below (n = 2, 0.19 and 0.21 mg/L) or marginally within (n = 4, 0.31–0.51 mg/L) the reference range. Serum albumin and total protein were normal in 16 of 17 dogs but reduced in one affected dog (Table S1). The vitamin A levels were thus directly correlated with immunoreactive RBP across genotypes (*r*^2^ = 0.88; Figure 4D). Collectively, these results suggest that the K12del mutation destabilizes RBP *in vivo*, preventing mobilization of vitamin A from maternal liver stores to the embryo. Moreover, misfolded K12del dimers are unlikely to interact effectively with retinol, transthyretin, or STRA6 *in vivo*, given the behavior of human pathogenic *RBP4* missense alleles *in vitro* (Chou et al., 2015) and steric constraints evident in the X-ray structure of *holo* RBP-TTR_4_ (Berni and Formelli, 1992). However, formally the possibility of the direct binding of the K12del mutant to STRA6 could be tested, as described previously (Chou et al., 2015).

Mutant RBP dimers may be cleared from the bloodstream by megalin (LRP2) or other receptors (Wyatt et al., 2011), but they are unlikely to enter the urine in the absence of renal damage. In normal mammals, *holo* RBP circulates bound to TTR tetramers, which increases its effective molecular weight (>75 kDa) and prevents filtration in the kidneys (Vahlquist et al., 1973). To test this hypothesis, we measured RBP levels in urine (uRBP) samples from the 17 genotyped dogs whose serum data are described above. We detected uRBP in only two samples, a K12del homozygote and a K12del/+ carrier, at approximately 1/20^th^ the mean WT serum level (Figure S4). These two dogs are likely to have impaired renal function, as total urinary protein was also elevated (data not shown).

### The K12del Mutation Impairs Secretion of RBP Monomers from Cultured HeLa Cells

**Figure 5:**
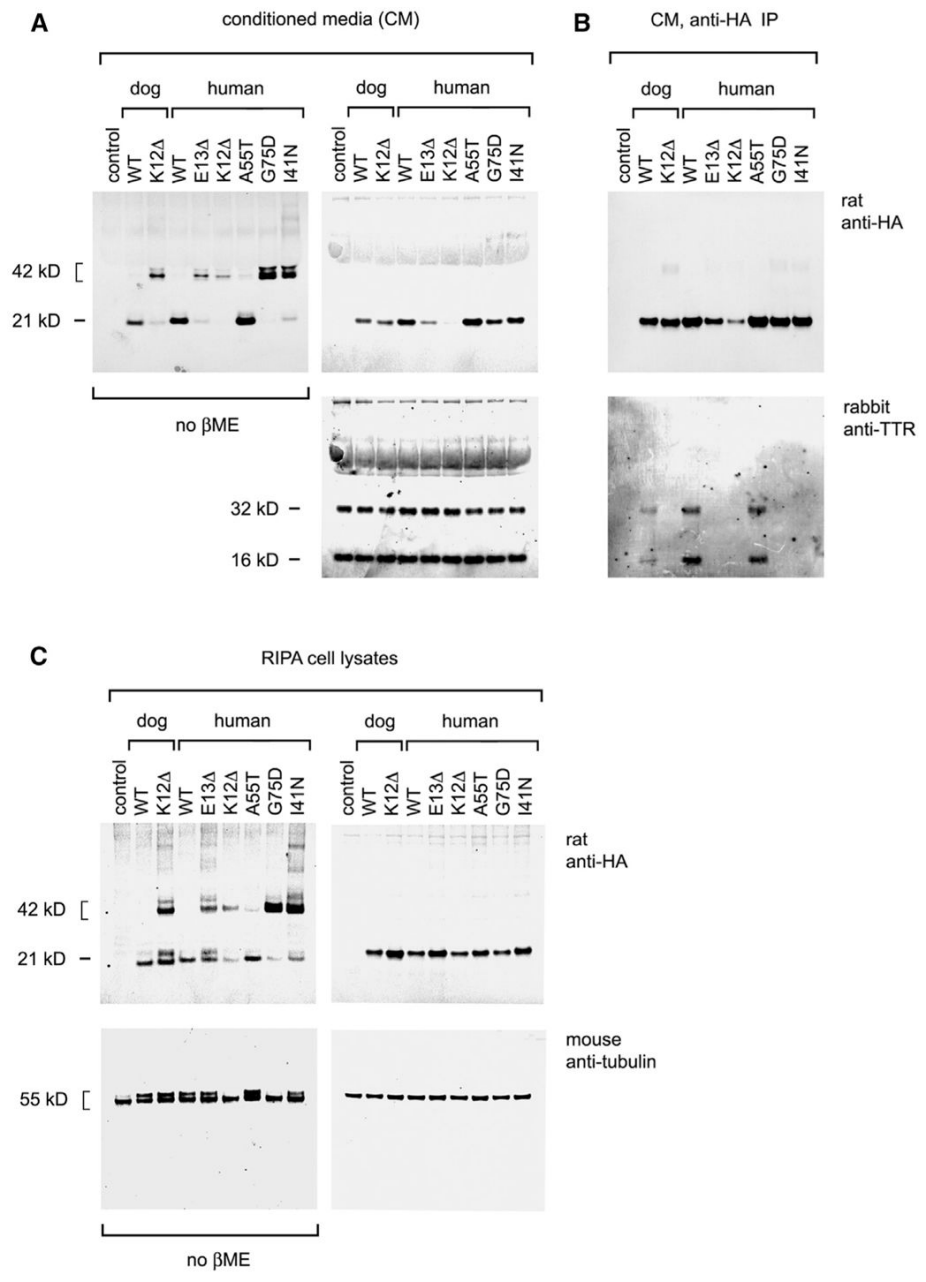
RBP Secretion and Transthyretin Binding *In Vitro* (A–C) LI-COR western analysis of (A) CM, (B) immunoprecipitates (IP) of CMs, and (C) radio-immunoprecipitation assay buffer (RIPA) lysates from transfected HeLa cells expressing dog (WT and K12del) or human (WT, K12del, E13del, A55T, G75D, and I47N) ^HA^RBPs, detected using anti-HA antibody. In (A) and (B), parallel gels were electrophoresed under reducing (+βME, right) or non-reducing conditions (no βME, left), with bovine TTR and human α-tubulin as CM and lysate loading controls, respectively. (A) Recombinant ^HA^RBPs are secreted as dimers or higher multimers (K12del in both species; human E13del, G75D, and I41N) and monomers (WT in both species, human A55T). The K12del dimers secreted by HeLa cells *in vitro* appear similar to RBP dimers secreted by mutant dogs *in vivo*, and presumably they reflect the exposure of unpaired cysteines, which persist after intra-molecular oxidative folding. The A55T, G75D, and I41N proteins behave as previously reported (Chou et al., 2015). (B) The RBP profiles of transfected cell lysates and CMs generally correspond; however, mutant monomers are notably more abundant (>50%) in cell lysates than in CMs. These abnormal RBPs are presumably retained in the ER and secreted following dimerization. (C) Co-immunoprecipitation showing that only WT and stable A55T mutant ^HA^RBPs interact with bovine TTR present in the CMs. See also Figure S3.

As a further test of K12del effects, we compared the abundance and structure of RBP polypeptides secreted into the conditioned media (CMs) and retained in the cytoplasm of transfected HeLa cells and their interactions with transthyretin (Figure 5). Plasmid constructs expressing K12del or WT canine RBP or orthologous human mutants (K12del or E13del) were generated (Table S3) and tested in parallel, with human WT and mutant controls, including stable (A55T) and unstable (G75D and I41N) pathogenic isoforms (Chou et al., 2015). Western analysis of CMs and cell lysates, performed under reducing and non-reducing conditions (±βME), showed that secretion of K12del (and E13del) mutants was significantly altered, with a striking predominance of dimers. The dimer fractions for K12del and WT canine RBP in CMs were 0.89 and 0.007, respectively (Figure 5A, left), whereas the total amount of RBP secreted was similar (Figure 5A, right). Likewise, human E13del, K12del, G75D, and I41N mutants were secreted into CMs as >85% dimers, compared to <1% for A55T and WT controls, and secretion of human E13del and K12del was diminished. There were at least two distinct RBP dimer species in CMs, indicated by closely migrating 42-kDa products in the non-reducing western blot (Figure 5A). This conformational heterogeneity is likely to reflect the formation of intermolecular disulfide bonds between different cysteine pairs, with a variable degree of compactness. In a previous study of RBP oxidative folding in HepG2 cells in the presence of DTT, an ensemble of folding intermediates was similarly identified by their heterodisperse migration in non-reducing gels (Kaji and Lodish, 1993).

Western analyses of cell lysates ± βME further showed that dog K12del and human E13del mutant RBPs accumulated in cytoplasm, with normalized lysate-to-CM ratios of 1.5 and 6.0, respectively, compared to WT (ratio = 1.0) (Figure 5C, right). The abundance of RBP monomers in mutant cell lysates was notable, given their paucity in CMs, and these proteins migrated as ≥2 different species (Figure 5C, left). Monomers comprise >50% of RBP in lysates but <15% of RBP secreted into CMs by HeLa cells expressing dog K12del or human E13del mutants. In contrast, monomers comprise >99% of WT RBP in cell lysates and CMs. Together, these data suggest that the ISCWT mutation disrupts the kinetics of RBP folding *in vivo* and slows secretion, with iterative cycles of oxidative refolding or dimerization in the ER as a likely rate-limiting step (Ruggiano et al., 2014).

To further assess mutant RBPs, we tested their interaction with bovine transthyretin (TTR) in CM by immunoprecipitation (Figure 5B). In these experiments, WT and stable mutant RBPs bound TTR, but K12del and other mutants did not. The low levels of serum RBP in mutant dogs may thus arise from decreased hepatic secretion and increased renal or systemic clearance of abnormal RBP dimers.

### K12del Protein Can Fold as a Monomer and Bind Vitamin A In Vitro

To evaluate how the mutation alters RBP structure more precisely, we expressed recombinant WT and K12del proteins in *E. coli* strain Origami B(DE3), which has an oxidizing cytoplasmic environment allowing disulfide bond formation, and we used gel filtration (size exclusion chromatography [SEC]) as the final purification step (Kawaguchi et al., 2013). WT and K12del RBPs eluted in the same fraction (volume 92 mL) in parallel columns, indicating that both proteins have the same overall size and monomeric form (Figure S5). The chromatograms also showed small dimeric (elution volume 82 mL) and multimeric peaks, which were similar for both variants. The extent of aggregation depended on the concentration of purified proteins: both WT and K12del RBPs were monomeric in concentrations under 0.8 mM but aggregated at higher concentrations, as indicated by an increased nuclear magnetic resonance (NMR) line width.

**Figure 6:**
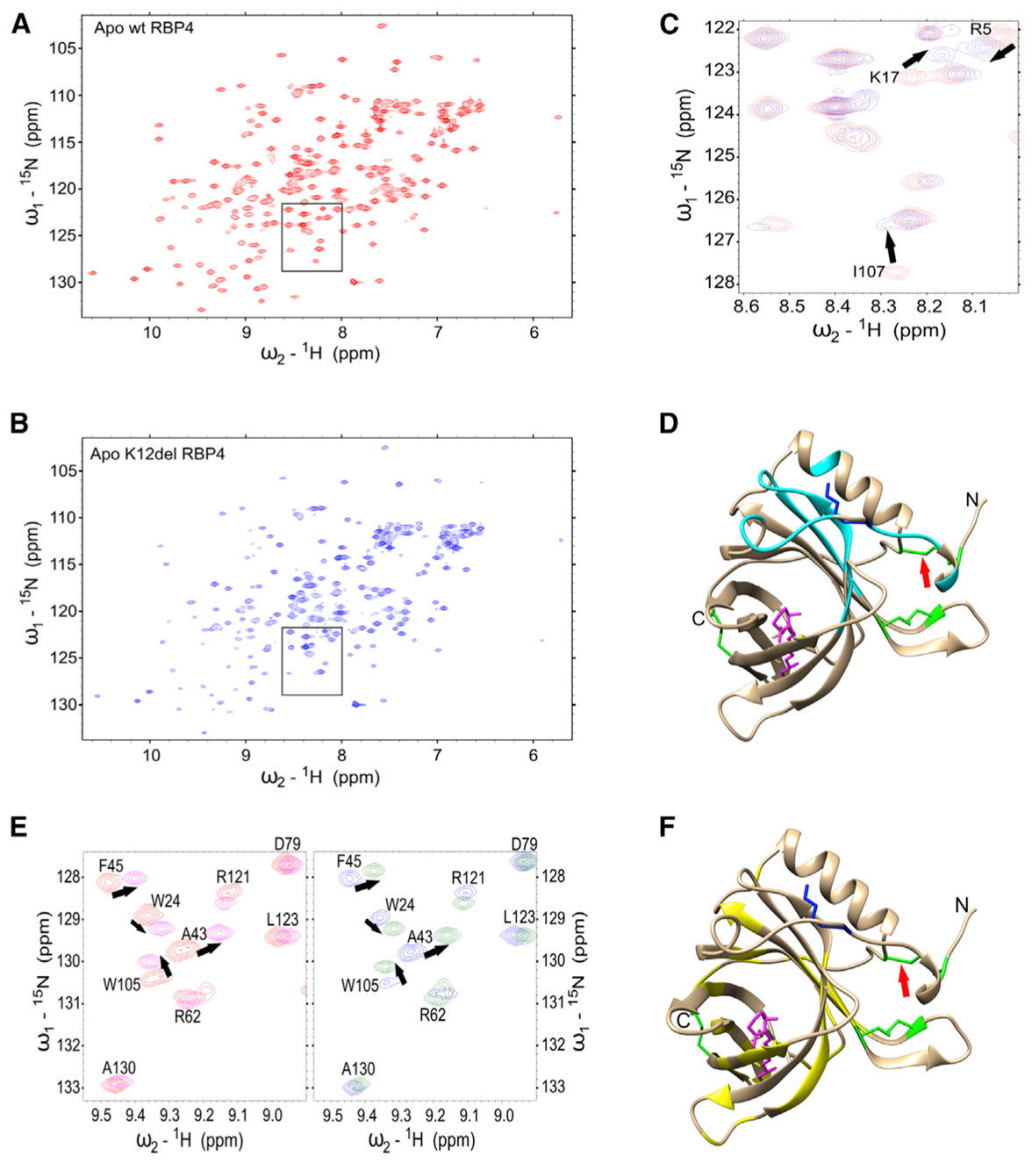
NMR Analysis of RBP Folding and Retinol Binding *In Vitro* (A and B) ^15^N-HSQC spectra of (A) *apo* WT RBP (red) and (B) *apo* K12del RBP (blue) produced in Origami B(DE3) *E. coli*. Both spectra are characteristic for native folded proteins. (C) Enlarged view of critical region (black box in A and B) with WT and K12del spectra overlaid for comparison. Large chemical shift perturbations (CSPs) observed for I107, K17, and R5 are denoted by black arrows. (D) Schematic ribbon diagram of WT canine *holo* RBP showing the largest chemical shifts changes caused by the K12del mutation (cyan). The K12 side chain (blue), disulfide bridges (green), C4–C160 disulfide bridge (red arrow), and all-*trans* retinol (vitamin A, magenta) are marked. The structure is modeled from human RBP coordinates obtained from PDB: 1BRP (Zanotti et al., 1993b; Mobli and King, 2010). (E) Representative region of ^15^N-HSQC spectra showing vitamin A binding to WT RBP (left panel, *apo* [red] versus *holo* [magenta]) and K12del RBP (right panel, *apo* [blue] versus *holo* [green]). The congruent patterns suggest that the retinol contact sites and binding affinities of WT and K12del RBP are similar. (F) Ribbon diagram showing the largest CSPs due to vitamin A binding (yellow) in both variants, with K12 and disulfide bridges marked as in (D). See also Figure S5.

A heteronuclear single quantum coherence (^15^N-HSQC) spectra of WT and K12del clearly showed that both variants folded *in vitro* and that the deletion did not significantly disrupt the overall structural integrity of RBP (Figures 6A–6D). As expected, chemical shift perturbations (CSPs) between WT and K12del were observed for residues that were spatially close to the deletion site (K12). Chemical shift of cysteine β-carbons is a reliable indicator of cysteine oxidation state (Sharma and Rajarathnam, 2000; Mobli and King, 2010). The observed cysteine β-carbon (Cβ) chemical shift values for residues C4 and C160 were 38.6 and 40.7 ppm, respectively, which are typical values for oxidized cysteine residue. The corresponding chemical shift values for reduced cysteines are 28.3 ± 2.2 ppm (Sharma and Rajarathnam, 2000). These data strongly suggest that C4 and C160 are oxidized and establish a disulfide bond within the recombinant K12del protein, similar to WT.

We also compared retinol binding of WT and K12del proteins using NMR spectroscopy. Retinol induced large CSPs for some residues in the ^15^N-HSQC spectrum of RBP, making their identification ambiguous, so we reassigned all chemical shifts for retinol-bound RBP (Greene et al., 2006). In this analysis, an equimolar ratio of vitamin A induced CSPs in the same residues of both RBP variants (Figures 6E and 6F). The K12 deletion thus does not alter the intrinsic retinol-binding mechanism or affinity, as WT and K12del RBPs synthesized *in vitro*-bound vitamin A similarly under the conditions studied.

Thus, whereas K12del RBP produced by canine hepatocytes *in vivo* or HeLa cells in culture is misfolded and secreted as abnormal dimers with little or no retinol cargo, NMR data clearly show that K12del RBP synthesized in a heterologous *E. coli* environment, outside the mammalian ER lumen and Golgi network, can fold properly at 16°C and form intra-molecular disulfide bridges similar to WT RBP and bind to vitamin A with the same interface and similar affinity as WT RBP (Figure 6F).

## DISCUSSION

In this study, we demonstrate that deletion of a single amino acid from the canine serum RBP causes severe congenital eye malformations in ISCWTs using segregation, clinical, and molecular data. The phenotype is transmitted as an autosomal recessive trait with penetrance determined by the maternal genotype. This unusual inheritance pattern is caused by the disruption of vitamin A transport from maternal hepatic stores to the developing fetal eye, in situations where functional RBP is absent on *both* sides of the placenta. In WT mammals, RBP transfers fat-soluble retinol bidirectionally, to (influx) *and* from (efflux) cells at the materno-fetal interface, respectively, via the STRA6 receptor (Kawaguchi et al., 2012). The resulting deficiency of vitamin A in affected ISCWT embryos presumably limits RA signaling during critical stages of eye development. Two hits, maternal *and* fetal homozygous *RBP4* mutations, are evidently needed to reduce vitamin A levels below the threshold for phenotypic expression in offspring. A genetically similar recessive maternal effect has been noted in *Rbp4*-knockout mice, but only when the dam is maintained on a VAD diet after conception (Quadro et al., 2005), and it is predicted for human *RBP4*-null alleles (Biesalski et al., 1999; Khan et al., 2017) in the unlikely scenario where both mother and child are homozyotes. Maternally skewed expression of *RBP4* defects has been reported in human MAC pedigrees, but with dominant transmission and incomplete penetrance (Chou et al., 2015).

The difference in severity of *Rbp4* and *Stra6* phenotypes between species has been puzzling. In particular, the relatively mild developmental effects observed in mutant mice (Quadro et al., 1999) compared to humans (Pasutto et al., 2007; Chou et al., 2015) has led some to assert that the RBP-STRA6 pathway is relatively unimportant for vitamin A homeostasis, apart from retinal physiology (Berry et al., 2013). These discordant phenotypes may reflect differences among mammals in placental anatomy and function, relative dependence on tonic RBP-mediated vitamin A transport versus postprandial delivery of retinyl ester in chylomicron particles (D’Ambrosio et al., 2011), or direct transfer of vitamin A via uterine lumenal secretions (Suire et al., 2001). The canine model confirms the importance of RBP and STRA6 for mobilizing vitamin A during fetal development and further illuminates this pathway. Notably, the ISCWT phenotype manifests without dietary restriction. While the exact cellular interface for materno-fetal vitamin A transfer is poorly defined (Marceau et al., 2007), the placentae of rodents and primates are hemochorial, with direct contact between maternal blood and trophoblast layers, whereas carnivores such as dogs have endotheliochorial placentae, with greater histocompartmental separation between maternal and fetal circulation (Wooding and Burton, 2008).

To fully understand the molecular effects of the ISCWT mutation, we analyzed K12del RBP4 at three levels: as recombinant protein purified from bacteria, as protein secreted in the CMs of cultured HeLa cells, and as canine sera *in vivo*. When K12del RBP is expressed in the Origami B(DE3) *E. coli* strain under favorable conditions at 16°C, in an oxidizing cytoplasm created by double thioredoxin (*trxB*) and glutaredoxin reductase (*gor*) mutations, it folds correctly, forms internal disulfide bonds, and binds retinol similar to WT, with altered residue positions but no obvious molecular strain. In contrast, when K12del RBP is expressed by eukaryotic cells, both canine hepatocytes and HeLa cervical carcinoma cells, it misfolds, such that one or more internal disulfide bonds does not form within the ER lumen, during or after translation and signal peptide cleavage, leaving unpaired cysteines. The misfolded polypeptides with exposed thiol groups are presumably retained and destroyed via the ERAD (ER-associated protein degradation) pathway, following abortive refolding cycles (Ruggiano et al., 2014), or they are linked to other misfolded RBPs via intermolecular cysteine disulfide bridge(s), passed by ER quality control (Vembar and Brodsky, 2008), and secreted as homodimers. During oxidative folding of WT RBP in ER microsomes, facilitated by multiple chaperones, the C120–C129 disulfide bond forms first and is most critical to stability; the large C4–C160 and C70–C174 loops form subsequently (Selvaraj et al., 2008). Assuming K12del and WT alleles are transcribed and translated with equal efficiency, our quantitative western analyses of dog sera suggest that most K12del protein is degraded prior to secretion or rapidly cleared from the bloodstream.

These disparate results are instructive. K12del folding must be *thermodynamically* favored, as it occurs readily, in a heterologous *E. coli* environment. However, the folding funnel must have altered topology, compared to WT (Dill and Chan, 1997). As noted by Cowan et al. in their original RBP X-ray crystal structure report, “residues which contribute to the formation of the retinol-containing barrel start at residue 12. The methylene groups of the lysine side chain of K12 help close off the barrel… “ (Cowan et al., 1990). Consequently, K12del folding within the mammalian ER lumen may be kinetically compromised; it proceeds too slowly to escape the ERAD pathway and allow secretion of K12del RBP monomers. Misfolding occurs in K12del mutant dogs *in vivo*, despite the presence of ample hepatic retinol, which acts as a molecular chaperone to stimulate correct RBP folding and co-secretion of the *holo* RBP-TTR complex (Kawaguchi et al., 2013; Bellovino et al., 1996).

We believe the eukaryotic data best explain the clinical findings and are most relevant and that the K12del mutation acts as a null allele. Indeed, WT *apo* RBP, expressed in reducing *E. coli* strains (e.g., BL21) and oxidized randomly *in vitro* with glutathione (GSSG/GSH), exists as an ensemble of conformers, including several products with non-native disulfide bonds, and it must be further purified for biochemical assays (Kawaguchi et al., 2013). Moreover, an engineered RBP expressed in *E. coli* with six cysteine-to-serine substitutions can fold correctly *in vitro* and bind vitamin A in the absence of stabilizing disulfide bonds (Reznik et al., 2003), whereas similar mutant RBPs expressed in isolated dog microsomes cannot (Selvaraj et al., 2008). Finally, in a previous study of recessive human *RBP4* alleles, G75D and I41N proteins expressed in *E. coli* were reported to fold correctly, bind TTR and retinol, with reduced stability of the *holo* RBP complex (Folli et al., 2005), yet these proteins were undetectable in patient serum (Biesalski et al., 1999) and are secreted as aggregates from transfected HeLa cells (Chou et al., 2015).

Together, our findings highlight a unique type of autosomal recessive inheritance in mammals, a maternal effect on penetrance with a physiological basis in nutrient transport across the placenta. Our study establishes a large animal model to investigate RBP4 function and pathology and enables novel treatment options.

## EXPERIMENTAL PROCEDURES

Further details and an outline of resources used in this work can be found in the Supplemental Experimental Procedures.

### The Canine Study Cohort

The study cohort was established from privately owned purebred ISCWTs with their owners’ consent and included 17 cases (11 males and 6 females) from 4 closely related litters and 23 controls (9 males and 14 females). All dogs were 6–10 weeks old at the time of clinical examinations, as described in the Supplemental Experimental Procedures. DNA analysis was also performed on 254 ISCWTs (111 males and 143 females) of different ages to evaluate the carrier frequency of the mutation in the breed. Sample collection and clinical studies were performed with approval from the Animal Ethical Committee of the County Administrative Board for Southern Finland (ESAVI/6054/04.10.03/2012), and all experiments were performed in accordance with relevant guidelines and regulations.

### Genetic Analyses

A GWAS and whole-genome sequencing were performed to map the disease locus and to identify the causative variant followed by a population screening by Sanger sequencing, as described in the Supplemental Experimental Procedures.

### Biochemical Studies

Serum and urine samples for RBP and vitamin A assays were collected from 17 dogs and vitamin A was measured. To assess the protein structure and function immunoblot analysis of native canine RBPs and recombinant ^HA^RBPs expressed by cultured HeLa cells as well as structural NMR studies in *E. coli* were performed. Details of the biochemical studies are described in the Supplemental Experimental Procedures.

### Statistical Methods

Parametric tests and exact n values are provided in the respective Experimental Procedures and Results sections. Statistical significance was evaluated in relation to a threshold p value of 0.05 (for GWAS after 100,000 permutations).

## Supplementary Material

1

2

## Figures and Tables

**Table 1:** Eye Phenotypes and Maternal Genotypes of RBP4 p.K12del Homozygous Offspring

Dam Genotype	Microphthalmia in del/del Offspring	
	Present	Absent
Homozygote (del/del)	17	1^a^
Carrier (del/+)	0	9
Unknown (del/−)	0	1
Total	17	11

Sire genotypes were del/+ for 12 microphthalmic dogs and del/− for 5 dogs. For non-microphthalmic dogs, sire genotypes were del/+ for 7 dogs and del/− for 4 dogs. The skewed distribution of maternal genotypes in this retrospective analysis is highly significant (p < 10^−6^, Fisher’s exact test, *df* = 1).

a Chorioretinal hypoplasia with normal globe size.

## Highlights

- Recessive RBP4 defect with maternal transmission causes congenital eye disease
- Disease manifests only if both the dam and the offspring carry homozygous mutation
- Mutation disrupts RBP4 folding in vivo and results in protein hypomorph
- Biochemical serum vitamin A deficiency leads to disrupted fetal eye development
