# Supplementary material for: Maternal Inheritance of a Recessive RBP4 Defect in Canine Congenital Eye Disease
Source: Cell Rep. Author manuscript; Available in PMC 2019 Jun 3. (PMC6546432; doi:10.1016/j.celrep.2018.04.118)
Supplement: 1 [file NIHMS973284-supplement-1.pdf]

Cell Reports, Volume *23*

Supplemental Information

Maternal Inheritance of a Recessive RBP4 Defect in Canine Congenital Eye Disease

Maria Kaukonen, Sean Woods, Saija Ahonen, Seppo Lemberg, Maarit Hellman, Marjo K. Hytönen, Perttu Permi, Tom Glaser, and Hannes Lohi

Supplemental information

Supplemental experimental procedures
Supplemental references
Supplemental figures 1–5
Supplemental figure legends
Supplemental tables 1–3

Supplemental Experimental Procedures

Resource Table

REAGENT or RESOURCE	SOURCE	IDENTIFIER
Antibodies		
Rabbit anti-human RBP4	Dako	A0040
Rat anti-HA	Roche	3F10
Donkey anti-rabbit	Amersham	NA934/5
Bacterial and Virus Strains		
Origami B(DE3) *E. coli*	Novogen	N/A
Biological Samples		
Peripheral blood samples (EDTA) from Irish Soft-Coated Wheaten Terrier dogs	This paper	N/A
Serum samples from Irish Soft-Coated Wheaten Terrier dogs	This paper	N/A
Urine samples from Irish Soft-Coated Wheaten Terrier dogs	This paper	N/A
Critical Commercial Assays		
GC-Rich System Polymerase	Roche	12140306001
Serum vitamin A measurement	Thermo Fischer	N/A
Total protein and albumin measurement	IDEXX	N/A
Gibson assembly	NEB	E2611S
Deposited Data		
Raw and analysed data	This paper	ENA, ID: XXX
Oligonucleotides		
RBP4 deletion sequencing, forward: GCTCACGGCGCGGTCCCC	This paper	N/A
RBP4 deletion sequencing, reverse: GTTGTGGCCGGGCGAGCTC	This paper	N/A
Recombinant DNA		
Canine wild type and deletion mutation RBP4 plasmid	Genscript	N/A
pUS2-HA-RBP4	Chou et al. 2015	N/A
pET15b	Novagen	N/A
Software and Algorithms		
PLINK 1.07	Purcell et al. 2007	http://zzz.bwh.harvard.edu/plink/
GenABEL	Aulchenko et al. 2007	http://www.genabel.org
Burrows-Wheeler Aligner 0.5.9	Li & Durbin 2009	https://sourceforge.net/projects/bio-bwa/files/
Picard tools		http://sourceforge.net/projects/picard
Genome Analysis Tool Kit 2.6	McKenna et al. 2010	https://software.broadinstitute.org/gatk/
TopSpin 3.5	Bruker	N/A
Sparky	Goddard and Kneller	N/A
Other		
Heidelberg Spectralis Optical Coherence Tomography instrument	Heidelberg Engineering GmbH	N/A
Illumina Canine HD BeadChip genotyping arrays	Illumina	
Illumina HiSeq2500 sequencer	Illumina	N/A
Avance III HD 800 MHz spectrometer	Bruker	N/A

Clinical studies
In the initial cohort of 40 ISCWTs, the eyes of all dogs were examined by veterinary ophthalmologists before 10 weeks of age, in order to fully evaluate CEA features in the fundus, as retinal pigmentation can mask this phenotype in older dogs (Bjerkås 1991). Exams included a basic neuro-ophthalmic assessment, slit-lamp biomicroscopy to evaluate the anterior segment and adnexa, and indirect ophthalmoscopy to evaluate the fundus. Topical tropicamide (Oftan Tropicamid 1%, Santen, Tampere, Finland) was used to achieve mydriasis. Optical coherence tomography (OCT) was performed on 11 ISCWTs with a Heidelberg Spectralis instrument (Heidelberg Engineering GmbH, Germany). The inclusion criteria for cases was severe bilateral microphthalmia. DNA analysis was performed on 254 ISCWTs, including the dams (mothers) of affected litters and their dams (maternal grandmothers) to determine *RBP4* genotypes. Of these dogs, 175 were examined as adults by veterinary ophthalmologists as described above.

DNA extraction
Peripheral blood samples (3 ml) for DNA studies were collected in EDTA tubes. Genomic DNA was extracted from white blood cells using a semi-automated Chemagen extraction robot (PerkinElmer Chemagen Technologie GmbH, Baeswieler, Germany), measured using a Qubit fluorometer (Thermo Fisher Scientific, Waltham, MA, USA) or Nanodrop ND-1000 UV/Vis spectrophotometer (Nanodrop technologies, Wilmington, DE, USA) and stored at –20 °C.

Genome wide association analysis
A genome-wide association study (GWAS) was performed on 12 cases and 17 controls using Illumina CanineHD BeadChip arrays with 172,963 markers (San Diego, CA, USA). Genotyping was performed by GeneSeek Laboratory (Neogen Genomics, Lincoln, NE, USA) and the results were filtered for quality control. Only SNPs conforming to Hardy-Weinberg expectations, with a genotyping rate >95% and minor allele frequency (MAF) >5%, were included in the analysis, leaving 91,542 informative SNPs in all. Allele frequency differences between case and control groups were evaluated for each SNP, with correction for multiple tests, and analyzed genome-wide using PLINK 1.07. Results were confirmed by GenABEL with full a genomic kinship matrix to adjust for population structure using mixed model approximation analysis (Purcell et al. 2007, Aulchenko et al. 2007).

Whole genome sequencing
Whole genome sequencing (WGS) was performed on one affected dog using the Illumina HiSeq2500 platform with 15X approximate coverage (paired-end reads, 2 x 100 bp). All reads were mapped to the dog reference genome (assembly CanFam 3.1) using the Burrows-Wheeler Aligner (BWA) version 0.5.9 (Li & Durbin 2009). Duplicate reads were marked and data sorted using Picard tools (http://sourceforge.net/projects/picard). Local alignment, BAM file generation and variant calling were performed using the Genome Analysis Tool Kit (GATK) version 2.6 (McKenna et al. 2010). Exonic variants were filtered using an in-house pipeline, assuming a recessive mode of inheritance for the ISCWT microphthalmia trait, and control data from 342 dogs of various breeds lacking this trait, which were collected from unrelated genome and exome sequencing projects (Table S2). Sanger PCR sequencing was used to confirm the *RBP4* variant found in WGS data and genotype the full ISCWT cohort.

Sanger PCR genotyping
RBP4 genotypes were determined Sanger sequencing. Forward (5'-GCTCACGGCGCGGTCCCC) and reverse (5'-GTTGTGGCCGGGCGAGCTC) primers flanking exon 2 were designed using Primer3 (Koressaar, Remm 2007), and products were amplified using GC-RICH System polymerase (Roche Diagnostics GmbH, Mannheim, Germany). Sanger cycle sequence data were analysed using Sequencher 5.1 (Gene Codes, Ann Arbor, MI, USA). As the ISCWT mutation destroys an *Xmn*I restriction site (GAANN^NNTTC), *RBP4* genotypes can also be determined by digesting the PCR products.

Serum retinol, albumin and total protein measurements
Serum and urine samples for RBP and VA assays were collected from 17 dogs. Vitamin A was measured by U-HPLC chromatography using a Dionex Instrument Rapid Separation LC 3000 unit with 325-nm diode array detector (Thermo Fisher Scientific, Waltham, MA, USA), after protein precipitation. Total protein and albumin were measured by IDEXX Laboratories (Hoofddorp, Netherlands) using an AU5800 Clinical Chemistry System analyser (Beckman Coulter, Brea, CA, USA).

Western analysis and immunoprecipitation
RBP levels were measured in canine serum (2 μL) or urine (10 μL) samples, and HeLa conditioned media (15 μL) or lysates (20 μg) by Western blot analysis, following polyacrylamide gel electrophoresis (SDS-PAGE). All dog samples were transported and stored at -80°C after collection. Total urinary and cell lysate protein levels were assayed by the Bradford method, using commercial reagents and bovine serum albumin (BSA) standards (Thermo Fisher). Aliquots were denatured for 10 min at 95°C in 1X loading buffer (0.5% lithium dodecyl sulfate, 2.5% glycerol, 12 mM EDTA, 62 mM Tris pH 8.5) and electrophoresed through 4-12% polyacrylamide Bis-Tris gels (NuPAGE, Invitrogen, Carlsbad,

CA, USA) in 2-(N-morpholino) ethanesulfonate (MES) running buffer pH 7.3 with 0.1% SDS, for 120 min at 25 V/cm. For analysis under reducing conditions, dithiothreitol (100 mM DTT) or 2-mercaptoethanol (100 mM βME) was added to samples before heating. Protein gels were electrotransfered to nitrocellulose membranes (GE Life Sciences, Piscataway, NJ, USA) in a Trans-Blot chamber (Biorad, Hercules, CA, USA) for 30 V x 16 h at 4°C in NuPAGE transfer buffer (25 mM Bicine, 25 mM Bis-Tris, 1 mM EDTA, pH 7.2) with 10% methanol. To fully mobilize proteins, expose epitopes and maximize detection of native RBP, nonreducing gels were soaked in 1X MES running buffer with 300 mM βME 1% SDS for 60 min at 25°C prior to transfer (Zettersrom, Stewart 2007).

For ECL (enhanced chemiluminescence) detection, membranes were washed in Tris-buffered saline (TBS), blocked in TBS 5% BSA 1% nonfat dry milk, and incubated for 16 h at 4°C with primary antibody. For infrared fluorescence detection, membranes were washed in TBS, blocked in Odyssey blocking buffer (OBB, LI-COR, Lincoln, NE, USA), and incubated with primary antibody in 1:1 mixture of OBB and 0.05% Tween-20 TBS. The primary antibodies were rabbit anti-human RBP4 (1:5000, Dako A0040, Carpinteria, CA, USA), rat anti-HA (1:5000, high affinity monoclonal 3F10, Roche), rabbit anti-human TTR (1:5000, Abnova PAB 1221, Walnut, CA, USA) and mouse anti-alpha tubulin (1:2000, monoclonal TU-01, Thermo Fisher Scientific, Waltham, MA, USA). For ECL detection, membranes were rinsed in TBS 0.05% Tween-20 (TBST), incubated at 25°C for 1 h with HRP (horseradish peroxidase)-conjugated donkey anti-rabbit IgG secondary antibody (1:5000, NA934, GE Amersham, UK) in TBS 5% BSA, and developed using ECL Plus reagents and X-ray film (GE Amersham). For dual wavelength infrared detection, membranes were co-incubated with IR 800CW- and 680LT-conjugated anti-rat, rabbit or mouse IgG secondary antibodies (1:20,000, LI-COR). In these experiments, the anti-rabbit secondary antibodies reacted weakly with dog IgG, which is relatively resistant to reduction (Singh, Whitesides 1994). RBP levels were quantified by densitometry of serial exposures (ImageJ, NIH, Bethesda, MD, USA), averaging signal intensity among ≥4 sample replicates, and normalizing results to wild-type mean.

Native ^{HA}RBP complexes were immunopurified from CM using mouse anti-HA monoclonal IgG conjugated agarose beads (A2095, Sigma-Aldrich, Saint Louis, MO, USA) and fritted spin columns. CM samples (0.5 mL) were incubated with beads for 16 h at 4°C and bound proteins were eluted in 200 μL 1X loading buffer for 15 min at 95°C. The interaction between secreted ^{HA}RBPs and bovine TTR in the overlying DMEM (10% FBS) was assessed by eluate Western blots (Chou *et al*. 2015).

Molecular cloning

The secreted portions of wild type and K12del canine *RBP4* cDNAs (accession XM_534969, GenScript, Piscataway, NJ, USA) were subcloned into pUS2 vector, downstream of the human *RBP4* signal peptide (MKWVWALLLLAALGSGRA) and HA epitope tag (YPYDVPDYA), by PCR and Gibson assembly (Gibson *et al*. 2009) with complementary primers (Table S3). Orthologous human K12del and E13del pUS2-RBP^{HA} expression plasmids (Chou et al. 2015) were generated by site-directed mutagenesis (Liu, Naismith 2008). All plasmids were verified by DNA sequencing.

Mammalian cell transfection and lysis

HeLa cells were cultured in Dulbecco's Modified Eagle Medium (DMEM) supplemented with 10% fetal bovine serum (FBS), 2 mM glutamine and 100 U/mL penicillin/streptomycin, at 37°C in a humidified 10% CO_2 atmosphere. Subconfluent cultures were transfected with 6 μg plasmid DNA and 6 μl of Xtremegene HP reagent (Roche) per 100 mm dish. Sixteen hours later, cells media was replaced with 5 mL DMEM. Conditioned media containing recombinant dog or human ^{HA}RBP was then harvested after an additional 48 h, filtered through 0.22 μm syringe units (Millipore, Billerica, MA, USA) and stored at 4°C. Adherent cells were harvested simultaneously, washed in PBS and frozen. Cell pellets were then lysed in RIPA buffer (0.1% SDS, 1% NP40, 1% sodium deoxycholate, 150 mM NaCl, 1 mM EDTA, 25 mM Tris pH 7.5) with protease inhibitors (Complete cocktail, Roche) and centrifuged at 16000x*g* to recover lysate supernatants.

RBP expression in E. coli and purification for NMR studies

The cDNAs encoding WT or K12del canine RBP4 (residues 19-201, GenScript) were subcloned into the *Nde*I and *Xho*I sites of pET15b vector (Novagen, Billerica, MA, USA), downstream from the GB1 protein coding sequence (*Streptococcus sp*. protein G IgG-binding domain) and TEV (Tobacco Etch Virus) protease cleavage site.

^{13}C, ^{15}N-labeled WT and K12del proteins were produced in the Origami B(DE3) *E. coli* strain (Novogen) to allow formation of disulfide bridges in the cytoplasm (De Marco 2009). Bacteria were grown in M9 minimal media, supplemented with 1 g/L $^{15}NH_4Cl$ as the sole nitrogen source, or with 1 g/L $^{15}NH_4Cl$ and 2 g/L ^{13}C-D-glucose as the sole nitrogen and carbon sources, respectively. Cultures were incubated at 37°C until an optical density (OD) of 0.4 was reached, and at 16°C thereafter. Protein production was induced by addition of 1 mM isopropyl β-D-1-thiogalactopyranoside (IPTG) when an OD of 0.6 was reached. Cultures were further incubated at 16°C for 16 h and collected by centrifugation. Cells were disrupted by sonication and the resulting supernatant was clarified by centrifugation at 30000x*g*.

Clarified supernatants containing GB1-RBP4 polypeptide were applied to 1-mL His GraviTrap columns (GE Healthcare, Wilmington, MA, USA) and eluted according to manufacturer instructions. Eluted fusion proteins were dialyzed extensively against PBS, digested with TEV protease, and applied to His GraviTrap columns to remove the polyHis-GB1 N-terminus. Cleaved RBP4 eluted in the flow-through fraction, which was then concentrated and applied to a HiLoad 16/60 Superdex 200 gel filtration column equilibrated 50 mM NaCl, 20 mM $NaPO_4$ pH 6 (NMR buffer). Fractions containing purified RBP were pooled and concentrated to 0.8 mM (16.8 mg/mL). Gel filtration was performed using the ÄKTA Purifier FPLC system (GE Healthcare).

NMR spectra were acquired at 308 °K using a Bruker Avance III HD 800 MHz spectrometer (Bruker, Billerica, Massachusetts, USA) equipped with a cooled 1H, 13C, 15N TCI cryoprobe. RBP samples were dissolved in NMR buffer containing 4% D_2O. *holo* RBP was generated by exposing recombinant RBP to 1 μM all-*trans* retinol in NMR buffer. Transverse relaxation optimized spectroscopy (TROSY) based, HN-detected triple resonance experiments HNCACB, CBCA(CO)NH, HNCA and HNCOCA (Muhandiram, Kay 1994, Sattler, Schleucher & Griesinger 1999, Permi, Annila 2004) were used to assign chemical shifts. NMR data were processed using TopSpin 3.5 (Bruker, Billerica, Massachusetts, USA) and analyzed with Sparky software (Goddard and Kneller, University of California, San Francisco).

Supplemental References

Aulchenko, Y.S., Ripke, S., Isaacs, A. and van Duijn, C. (2007). GenABEL: an R library for genome-wide association analysis. Bioinformatics *23*, 1294–1294.

De Marco, A. (2009). Strategies for successful recombinant expression of disulfide bond-dependent proteins in Escherichia coli. Microb. Cell Fact. *8,* 26.

Gibson, D.G., Young, L., Chuang, R., Venter, J.C., Hutchison, C.A. and Smith, H.O. (2009). Enzymatic assembly of DNA molecules up to several hundred kilobases. Nat. Methods *6,* 343–345.

Koressaar, T. and Remm, M. (2007). Enhancements and modifications of primer design program Primer3. Bioinformatics *23,* 1289–1291.

Li, H. and Durbin, R. (2009). Fast and accurate short read alignment with Burrows-Wheeler transform. Bioinformatics *25,* 1754–1760.

Liu, H. and Naismith, J.H. (2008). An efficient one-step site-directed deletion, insertion, single and multiple-site plasmid mutagenesis protocol. BMC Biotechnol. *8,* 91.

McKenna, A., Hanna, M., Banks, E., Sivachenko, A., Cibulskis, K., Kernytsky, A., Garimella, K., Altshuler, D., Gabriel, S., Daly, M. et al. (2010). The Genome Analysis Toolkit: a MapReduce framework for analyzing next-generation DNA sequencing data. Genome Res. *20,* 1297–1303.

Muhandiram, D. and Kay, L.E. (1994). Gradient-enhanced triple-resonance three-dimensional NMR experiments with improved sensitivity. J. Magnetic Resonance, Series B *103,* 203–216.

Permi, P. and Annila, A. (2004). Coherence transfer in proteins. Prog. Nucl. Magn. Reson. Spectrosc. *44,* 97–137.

Purcell, S., Neale, B., Todd-Brown, K., Thomas, L., Ferreira, M.A., Bender, D., Maller, J., Sklar, P., De Bakker, P.I. and Daly, M.J. (2007). PLINK: a tool set for whole-genome association and population-based linkage analyses. Am. J. Hum. Genet. *81,* 559–575.

Sattler, M., Schleucher, J. and Griesinger, C. (1999). Heteronuclear multidimensional NMR experiments for the structure determination of proteins in solution. Prog. Nucl. Magn. Reson. Spectrosc. *34,* 93–158.

Supplemental Figure 1

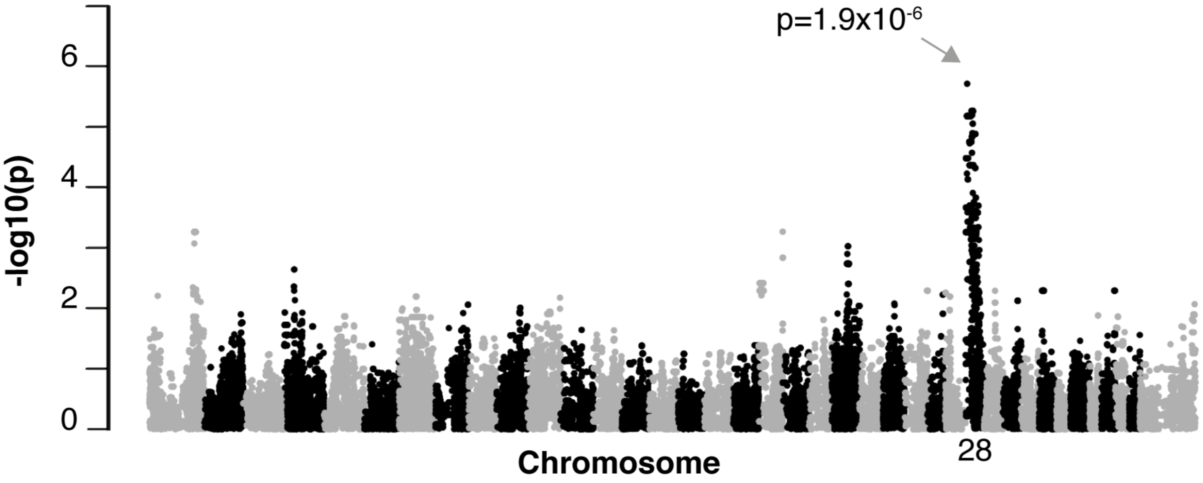

Supplemental Figure 1, related to Figure 2. GenABEL analysis of ISCWT genotype data, performed using a full genomic kinship matrix to adjust for population structure and mixed model approximation, reveals the MAC disease locus on the CFA28.

Supplemental Figure 2

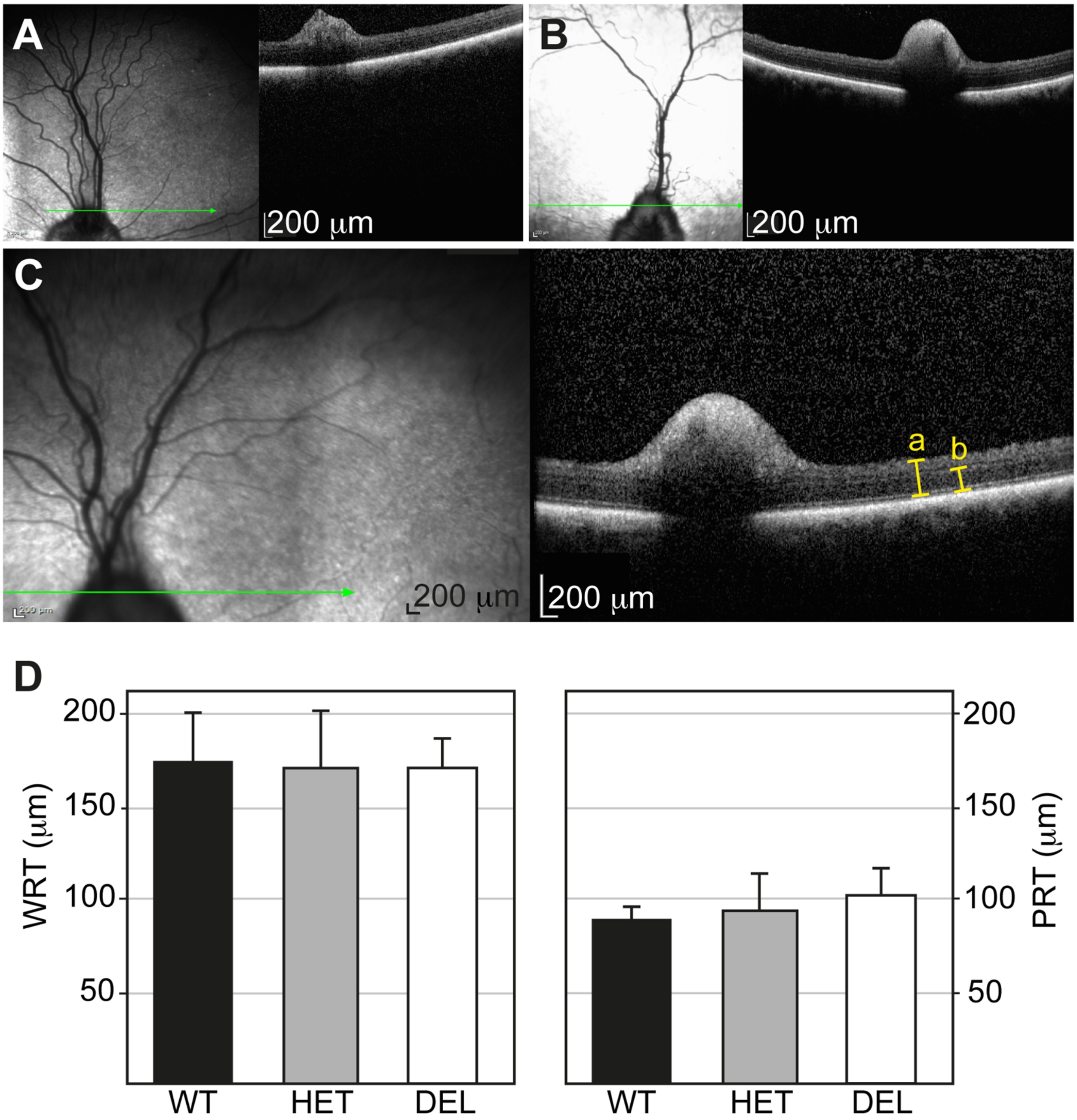

Supplemental Figure 2, related to Figure 1. Optical coherence tomography (OCT) assessment of whole retinal (WRT, *a*) and photoreceptor layer (PRT, *b*) thickness. **[A-C]** Representative fundus (*en face*, left) and OCT (transverse, right) views of WT (*n* = 3 dogs, 3 eyes) **[A]**, carrier (*n* = 4 dogs, 6 eyes) **[B]** and mutant retinas (*n* = 4 dogs, 6 eyes) **[C]** in the peripapillary tapetal region, with optic nerve head (elevated) and dorsal retinal vein landmarks. OCT section planes (green lines in fundus images) and measurement parameters (yellow brackets) are indicated. **[D]** Histograms comparing WRT and PRT values (µm ± SD) for *RBP4* genotype groups. The mean WRT was 175 ± 28 µm in wild type dogs, 173 ± 32 µm in carriers and 171 ± 16 µm in deletion homozygotes, and the mean PRT values were 89 ± 8 µm, 93 ± 22 µm and 100 ± 15 µm, respectively. The WRT and PRT values did not differ significantly (pairwise *t*-tests) between deletion homozygotes and wild type dogs (p = 0.8 and 0.2) or carriers and wild type dogs (p = 0.9 and 0.7), respectively.

Supplemental Figure 3

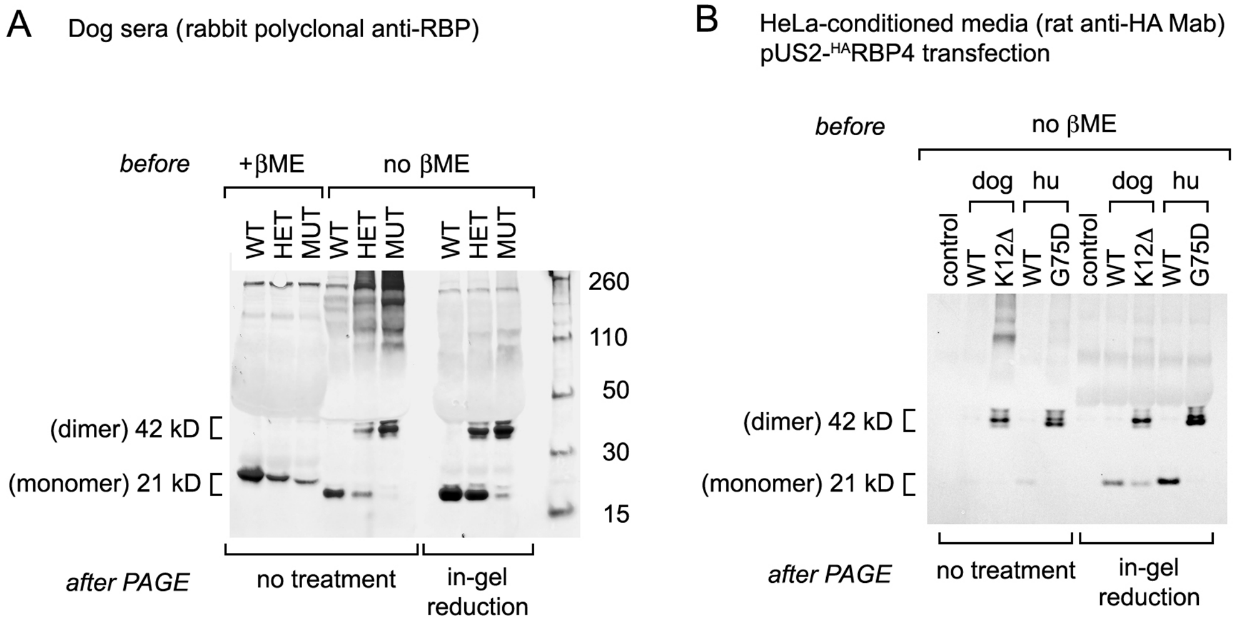

Supplemental Figure 3, related to Figure 5. In-gel reduction analysis of WT and mutant RBPs. **[A]** Western blot of +/+ (WT), del/+ (HET) and del/del (MUT) dog sera treated with βME before or after SDS polyacrylamide gel electrophoresis (PAGE). In the absence of βME, misfolded K12del dimers appear more antigenic than wild type globular RBP. In-gel reduction (Zetterstrom *et al.* 2007) enhances the wild type RBP signal. **[B]** Western blot of HeLa CM. Pre-transfer in-gel βME treatment increases exposure of the N-terminal HA epitope and improves detection of wild type ^{HA}RBP monomers.

Supplemental Figure 4

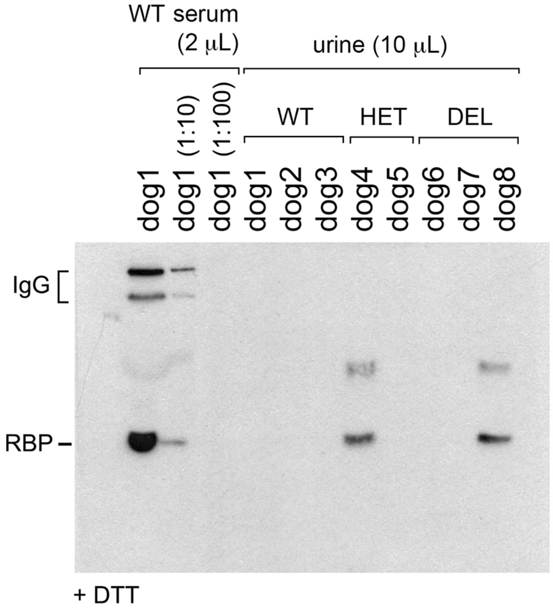

Supplemental Figure 4, related to Figure 4. Urinary RBP. Western blot showing detectable RBP in samples from two dogs, with serum dilutions for comparison. One carrier had obvious renal damage (urinary protein >3 mg/mL).

Supplemental Figure 5

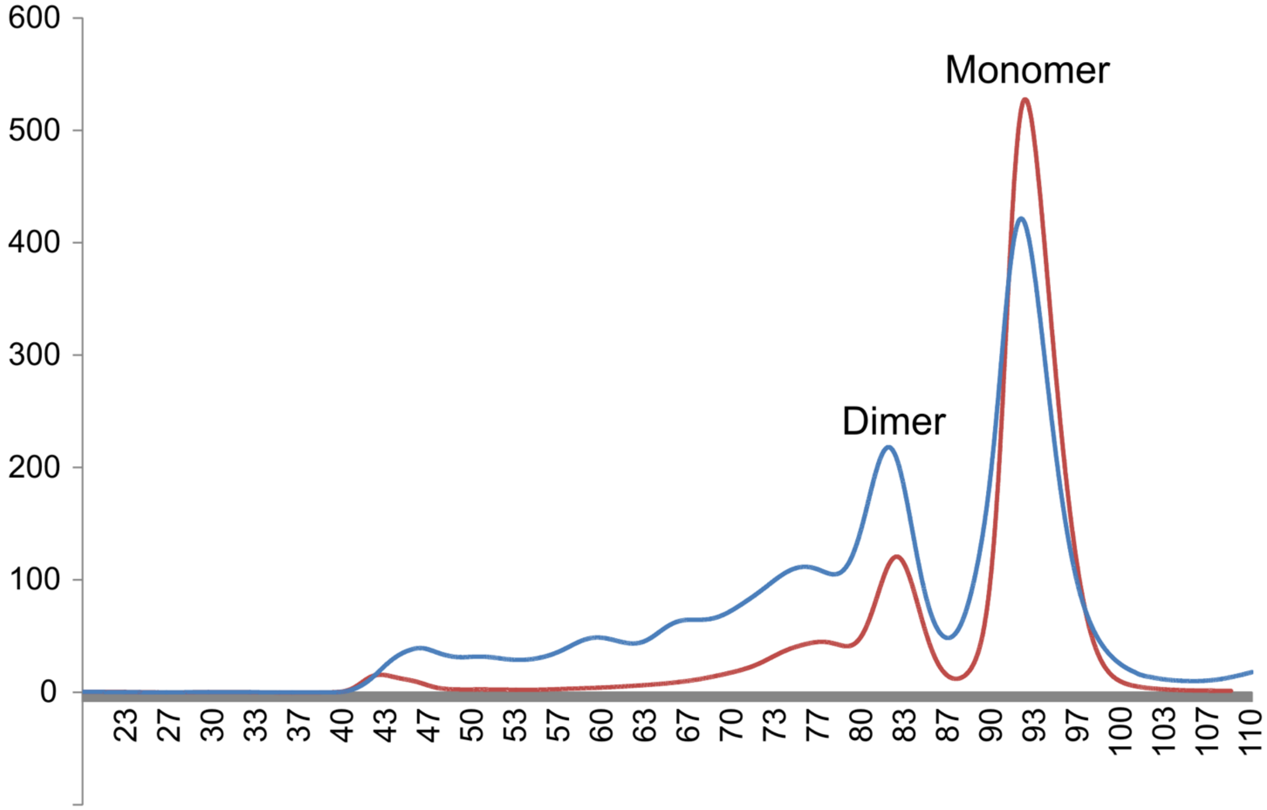

Supplemental Figure 5, related to Figure 6. Size exclusion chromatograms for recombinant (red) wild-type and K12del RBP (blue), produced as GB1-TEV-RBP fusion proteins in *E. coli* Origami B(DE3) and cleaved by TEV protease. The elution profiles (UV absorbance versus mL volume) are similar for both variants.

Supplemental Tables

Supplemental Table 1, related to Figure 1. Serum vitamin A, albumin and total protein measurements.

Supplemental Table 1. Serum vitamin A, albumin and total protein measurements.

Dog ID	Mutation status	Age at examination (in years)	Phenotype	S-Vitamin A (0.3-1.3 mg/L)	S-Albumin (28-43 g/L)	S-Total protein (54-76 g/L)
dog1	WT	5,9	Normal	0,66	34,00	68
dog2	WT	2,2	Normal	0,67	36,00	67
dog3	WT	1,3	Normal	0,32	32,00	62
dog4	carrier	1,9	Normal	0,51	40,00	73
dog5	carrier	0,1	Normal, used as control	0,19	33,00	62
dog6	12Kdel	9,9	Normal	0,09	34,00	69
dog7	12Kdel	3,2	Normal	0,06	34,00	66
dog8	12Kdel	3,7	Normal	0,04	33,00	70
dog9	12Kdel	0,1	Bilateral microphthalmia and severe bilateral CEA (choroideal hypoplasia and retinal coloboma). Case.	0,05	33,00	61
dog10	12Kdel	0,1	Bilateral microphthalmia, severe bilateral CEA (choroideal hypoplasia and retinal coloboma). Absent ONH in OD. Case.	0,06	33,00	61
dog11	12Kdel	0,1	Bilateral microphthalmia and moderate bilateral CEA (choroideal hypoplasia and retinal coloboma). Case.	0,06	22,00	38
dog12	12Kdel	1,9	Normal	0,05	37,00	62
dog13	12Kdel	8,4	Normal	0,10	37,00	68
dog14	carrier	0,1	Normal, used as control	0,31	38,00	65
dog15	carrier	0,1	Normal, used as control	0,33	34,00	63
dog16	carrier	0,1	Normal, used as control	0,46	38,00	70
dog17	carrier	3,5	Normal	0,21	35,00	63

CEA = collie eye anomaly, ONH = optic nerve head, OD = oculus dexter

Supplemental Table 2, related to Figure 2. 342 NGS dogs used for filtering (numbers per breed).

Supplemental Table 2. 342 NGS dogs used for filtering (numbers per breed).

Breed	Number of dogs
Afghan Hound	1
Airedale Terrier	6
Akita	3
Alaskan Malamute	6
American Hairless Terrier	5
Australian Cattle Dog	2
Australian Kelpie	12
Australian Shepherd	3
Australian Terrier	13
Barbet	2
Beagle	1
Bearded Collie	7
Belgian Shepherd, Groenendael	6
Belgian Shepherd, Malinois	5
Belgian Shepherd, Tervueren	7
Berger Blanc Suisse	1
Bichon Frisé	7
Black Russian Terrier	4
Border Collie	28
Boston Terrier	1
Bouvier	4
Boxer	4
Central Asian Shepherd Dog	1
Chihuahua	2
Dachshund	3
Dalmatian Dog	5
Dandie Dinmont Terrier	1
Dobermann Pinscher	11
Elo	1
English Bulldog	1
English Springer Spaniel	1
Entlebucher Sennenhund	8
Eurasier	2
Finnish Hound	6
Finnish Lapphund	5
Finnish Spitz	8
Fox Terrier	3
French Bulldog	2
German Pointer	3
German Shepherd	2
German Wirehaired	1
Great Dane	16
Irish Terrier	1
Italian Greyhound	3
Karelian Beardog	9
King Charles Spaniel	2
Kromfohrländer	1
Kuvasz	4
Labrador Retriever	11
Lagotto Romagnolo	9
Lancashire Heeler	2
Landseer	2
Lapponian Herder	4
Leonberger	5
Miniature Pinscher	2
Miniature Schnauzer	2
Newfoundland Dog	4
Norwegian Elkhound	2
Norwegian Lundehund	1
Norwich Terrier	1
Parson Russel Terrier	5
Pinscher	4
Polski Owczarek Nizinny	3
Pomeranian	1
Portuguese Podengo	1
Portuguese Water Dog	2
Pyrenean Shepherd	1
Rhodesian Ridgeback Dog	4
Rottweiler	7
Saluki	3
Samoyeed	2
Schnauzer	5
Shetland Sheepdog	3
Siberian Husky	3
Sloughi	3
Spanish Water Dog	1
Swedish Vallhund	4
Tibetan Mastiff	3
Welsh Springer Spaniel	2
West Highland White Terrier	2
Whippet	4
White Swiss Shepherd Dog	3
Yorkshire Terrier	1
Sum	342

Supplemental Table 3, related to Figure 5. Canine *RBP4* cloning and site-directed-mutagenesis primers.

Supplemental Table 3. Canine RBP4 cloning and site-directed-mutagenesis (SDM) primers.

Gibson PCRs	Size (bp)	F/R	Primers [5'-]
dog RBP4 cDNA	567	F	GTTCCAGATTACGCCGAGAGCGACTGCCGAGTGAG
		R	ATCTAGAGGCTCGAGCTACAAAGTGTTTGGTTCTGATCT
pUS2-huRBP4^{HA} vector	4421	F	CTCGAGCCTCTAGATTCTGCAGCCCT
		R	GGCGTAATCTGGAACATCGTATGGGT

Red, HA epitope tag sequence. Blue, pUS2 vector sequence.

Mutant	Size (bp)	F/R	Primers [5'-]
huRBP4 K12del	4970	F	GCTTCCGAGTC---GAGAACTTCGACAAGGCTCGCT
		R	TGTCGAAGTTCTC---GACTCGGAAGCTGCTCACTC
huRBP4 E13del	4970	F	TTCCGAGTCAAG---AACTTCGACAAGGCTCGCTTC
		R	CTTGTCGAAGTT---CTTGACTCGGAAGCTGCTCAC
